# *Glycyrrhizae Radix* Methanol Extract Attenuates Methamphetamine-Induced Locomotor Sensitization and Conditioned Place Preference

**DOI:** 10.1155/2014/152063

**Published:** 2014-10-15

**Authors:** ZhengLin Zhao, Young Woo Kim, YuPeng Yang, Jie Zhang, Ji Yun Jung, Suchan Chang, Il Je Cho, FuBo Zhou, JunChang Zhao, Bong Hyeo Lee, Chae Ha Yang, Sang Chan Kim, RongJie Zhao

**Affiliations:** ^1^Department of Pharmacology, Mudanjiang Medical University, 3 Tongxiang Street, Aimin District, Mudanjiang 157011, China; ^2^Medical Research Center, College of Oriental Medicine, Daegu Haany University, Daegu 706-060, Republic of Korea; ^3^The Fourth Affiliated Hospital of Harbin Medical University, Harbin 150001, China

## Abstract

*Glycyrrhizae Radix* modulates the neurochemical and locomotor alterations induced by acute psychostimulants in rodents via GABAb receptors. This study investigated the influence of methanol extract from *Glycyrrhizae Radix* (MEGR) on repeated methamphetamine- (METH-) induced locomotor sensitization and conditioned place preference (CPP). A cohort of rats was treated with METH (1 mg/kg/day) for 6 consecutive days, subjected to 6 days of withdrawal, and then challenged with the same dose of METH to induce locomotor sensitization; during the withdrawal period, the rats were administered MEGR (60 or 180 mg/kg/day). A separate cohort of rats was treated with either METH or saline every other day for 6 days in METH-paired or saline-paired chambers, respectively, to induce CPP. These rats were also administered MEGR (180 mg/kg) prior to every METH or CPP expression test. Pretreatment with MEGR (60 and 180 mg/kg/day) attenuated the expression of METH-induced locomotor sensitization dose-dependently, and 180 mg/kg MEGR significantly inhibited the development and expression of METH-induced CPP. Furthermore, administration of a selective GABAb receptor antagonist (SCH50911) prior to MEGR treatment effectively blocked the inhibitory effects of MEGR on locomotor sensitization, but not CPP. These results suggest that *Glycyrrhizae Radix* blocked repeated METH-induced behavioral changes via GABAb receptors.

## 1. Introduction

The abuse of methamphetamine (METH) results in a number of serious public health problems owing to its strongly addictive nature and potent neurotoxicity [1]. Despite the urgent need for—and the great effort to develop—medical interventions for the prevention of METH abuse, to date no medications have been approved by the US Food and Drug Administration for the treatment of METH addiction [2].

The rewarding effects of an abused drug, such as METH, are a key motivational factor that sustains an individual's addiction to that drug. In animal research, the rewarding effects of drugs are typically manifested as two measurable behaviors: behavioral sensitization and conditioned place preference (CPP; [3]), and the mitigation of these two behavioral phenotypes is potentially considered identical to addiction reduction therapies. Indeed, agents with antiaddiction properties, such as naltrexone and acamprosate, inhibit the development of behavioral sensitization and CPP following repeated exposure to abused drugs in rodent models [4, 5].

It is commonly agreed that all addictive drugs, including METH, increase dopamine (DA) release in the nucleus accumbens (Nacc) and that this increase is responsible for the rewarding effects of drugs [6]. Although several types of neurons are located in the Nacc, GABAergic medium spiny projection neurons are dominant among the neuronal population [7], and the role of DA in this region is to inhibit these neurons [8]. Moreover, GABAergic interneurons in the ventral tegmental area (VTA) inhibit mesolimbic dopaminergic neurons via the activation of GABAb receptors, which act as an important mechanism underlying regulation of the dopaminergic neuronal firing rate [9]. Thus, it is apparent that mesolimbic GABAergic neurons are critically important during the development and mediation of drug addiction. Consistent with this notion, both clinical and preclinical studies have demonstrated that the selective GABAb receptor agonist baclofen effectively treats psychostimulant addiction [10].

Radix* of Glycyrrhizae uralensis* (*Glycyrrhizae Radix*,* G. radix*), an important tonic used in traditional oriental medicine for the replenishment and invigoration of deficient Qi and blood, is widely recommended for its life-enhancing properties, ability to treat various injuries and swelling, and role in detoxification [11]. Historically, most pharmacological studies investigating* G. radix* have focused on its anti-inflammatory and antioxidative actions [12, 13], but over the past decade, several reports have emphasized the neuropharmacological properties of* G. radix*. For example, Gruenwald [14] reported that extracts from* G. radix* exert sedative, analgesic, and anticonvulsant effects, and Shishkina et al. [15] showed that glycyrrhizic acid produces anxiolytic effects in rats via an increase in brain monoamine levels. Moreover, previous studies from our laboratory have demonstrated that an extract derived from* G. radix* suppresses cocaine-induced accumbal DA release via action on GABAb receptors [16] and prevents METH-induced hyperlocomotion in rats by inhibiting accumbal DA production [17].

Based on the views that dopaminergic and GABAergic neurons play a critical role in METH addiction and that agents with neuroprotective properties may treat this addiction, the current study evaluated the possible therapeutic effects of methanol extract from* G. radix* (MEGR) on METH addiction in rats using locomotor sensitization and CPP.

## 2. Materials and Methods

### 2.1. MEGR Preparation

**Figure 1:**
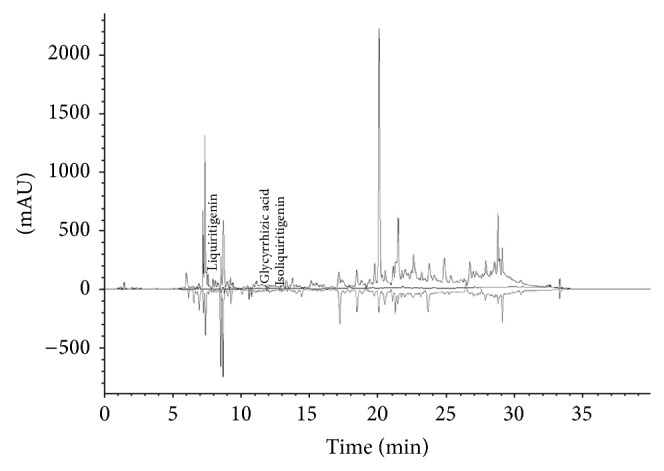
The HPLC profiles of MEGR at 254 nm (liquiritigenin), 276 nm (glycyrrhizic acid), and 380 nm (isoliquiritigenin). Flow rate: 1.0 mL/min, column: Waters XTerrat RP18 (150 × 4.6 mm, 5 *μ*m).

*G. radix* was purchased from Daewon Pharmacy (Daegu, Republic of Korea), and its identity and composition were confirmed by Professor Sang Chan Kim of the College of Oriental Medicine at Daegu Haany University in Korea. The MEGR was prepared by extracting 100 g powdered* G. radix* in 2 L methanol for 48 h, filtering the MEGR through a 0.2 *μ*m filter (Nalgene; NY, USA), and lyophilizing it in a vacuum evaporator. The MEGR was stored at −20°C until use. The amount of MEGR was estimated based on the dry weight of the lyophilized MEGR; the yield of the lyophilized MEGR was 18.36%. A high-performance liquid chromatography (HPLC) fingerprint of MEGR was developed following its dissolution in methanol (Figure 1); the lyophilized powder contained 357.10 ppM glycyrrhizic acid, 141.17 ppM liquiritigenin, and 41.01 ppM isoliquiritigenin. The standards for glycyrrhizic acid, liquiritigenin, and isoliquiritigenin were purchased from Sigma-Aldrich (St. Louis, MO, USA).

### 2.2. Animals

Male Sprague-Dawley rats (280–300 g) were obtained from Hyochang Science (Daegu, Korea) and acclimatized for 1 week prior to all experimental procedures. All rats were housed three per cage, provided with commercial rat chow (Purina; Seoul, Korea) and water* ad libitum*, and maintained in a filtered pathogen-free air environment between 21 and 23°C, at 50% relative humidity, and under a 12 h light/dark cycle. All experiments were conducted between 09:00 and 16:00 under standard conditions with controlled temperature, dim lighting, and low noise. All experimental procedures were conducted in accordance with the National Institutes of Health guidelines concerning the care and use of laboratory animals and were approved by the Animal Care and Use Committee of Daegu Haany University.

### 2.3. Locomotor Activity Test

Locomotor activity was assessed in a rectangular box (40 × 40 × 45 cm^3^) with floor and walls made of clear Plexiglas and painted black. The chamber was equipped with a video camera above the center of the floor, and all locomotor activity was monitored by a video tracking system using the Ethovision program (Noldus Information Technology BV; Wageningen, The Netherlands).

### 2.4. CPP Apparatus

The CPP apparatus was purchased from San Diego Ins (San Diego Instruments, San Diego, CA, USA) and consisted of three rectangular chambers separated by guillotine doors [18]. The center chamber (16 × 21 × 33 cm^3^) was connected by two end chambers that were identical in size (26 × 21 × 33 cm^3^) but distinguished by wall color and floor texture: one end chamber had black walls and a smooth floor (Chamber A), while the other had white walls and a textured floor (Chamber B). In the experimental condition, the rats showed a significant spontaneous preference for Chamber A, and thus a biased procedure was employed in which Chamber B was used as the METH-paired compartment. Animal movement and the time spent in each chamber were automatically recorded by a computer.

### 2.5. METH-Induced Locomotor Sensitization and Drug Treatment

**Figure 2:**
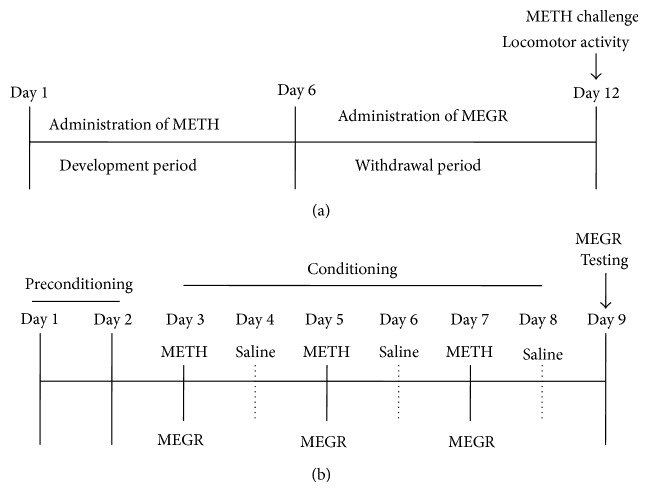
Time schedules for METH-induced locomotor sensitization and CPP. (a) Time schedule for locomotor sensitization: rats were treated with METH (1 mg/kg/day) for 6 consecutive days, subjected to 6 days of withdrawal, and then challenged with the same dose of METH to induce locomotor sensitization; during the withdrawal period, the rats were administered MEGR (60 or 180 mg/kg/day). (b) Time schedule for CPP: rats were treated with either METH or saline every other day for 6 days in METH-paired or saline-paired chambers, respectively, to induce CPP. These rats were also administered MEGR (180 mg/kg) prior to every METH or the CPP expression test.

All rats were administered intraperitoneal (i.p.) saline (1 mL/kg) or METH (obtained from the Korean Food and Drug Administration; 1 mg/kg, dissolved in saline) in their home cages for 6 consecutive days before undergoing 6 days of withdrawal. During the withdrawal period, the rats were orally administered distilled water (DW) or MEGR (60 or 180 mg/kg/day, dissolved in DW) once a day. Immediately after the final dose of DW or MEGR, the rats were adapted to the locomotor testing boxes for 60 min and then challenged with either METH (1 mg/kg) or saline. Following the challenge, the rats stayed in the boxes for an additional 60 min during which locomotor activity was assessed (Figure 2(a)) [19]. Additionally, to evaluate the possible involvement of GABAb receptors in MEGR-influenced expression of METH-induced locomotor sensitization, the selective GABAb receptor antagonist SCH50911 (3 mg/kg, dissolved in 5% Tween-80; Tocris Bioscience; Ellisville, MO, USA) was administered (i.p.) to the rats 10 min prior to MEGR treatment on the METH challenge day.

### 2.6. METH-Induced CPP and Drug Treatment

The CPP experiment consisted of three distinct phases: preconditioning (days 1 and 2), conditioning (days 3–8), and testing (day 9; Figure 2(b)). On day 1 of the preconditioning phase, each rat was placed in the center chamber of the CPP apparatus with the doors open and was allowed to freely explore both end chambers for 20 min. On day 2, the time spent in each chamber was recorded, and the chamber in which the rat spent more time was appointed as the preferred chamber (Chamber A) and the other chamber as the nonpreferred chamber (Chamber B). On days 3, 5, and 7 of the conditioning phase (acquisition phase), the rats were injected with METH (1 mg/kg) and confined to Chamber B for 60 min. On days 4, 6, and 8, the rats were administered saline and confined to Chamber A for 60 min. On day 9, for the testing phase (expression phase), each rat was again placed in the central chamber with the doors open, and the time spent in each end chamber was recorded for 20 min (Figure 2(b)). The change in place preference was calculated as follows:

(1) Change  in  place  preference  (Δsecond):Timetesting  phase  −Timepre-conditioning  phase (in  Chamber  B).

To evaluate whether MEGR inhibited the development and expression of METH-induced CPP, 180 mg/kg MEGR was administered to one cohort of rats 60 min prior to each METH administration during the conditioning phase or prior to the beginning of the testing phase. Additionally, SCH50911 (3 mg/kg) was administered to another set of rats 10 min prior to MEGR treatment on the testing day to examine the possible involvement of GABAb receptors in the MEGR-influenced expression of METH-induced CPP.

### 2.7. Statistical Analysis

All data were expressed as means ± standard errors of the mean (SEM) and analyzed by one-way analysis of variance (ANOVA) with a Newman-Keuls multiple comparison test or by a paired *t*-test (two groups) using the commercially available software GraphPad Prizm 5.0 (GraphPad Software; San Diego, CA, USA). A *P* value < 0.05 was considered statistically significant.

## 3. Results

### 3.1. Effects of MEGR on METH-Induced Locomotor Sensitization

**Figure 3:**
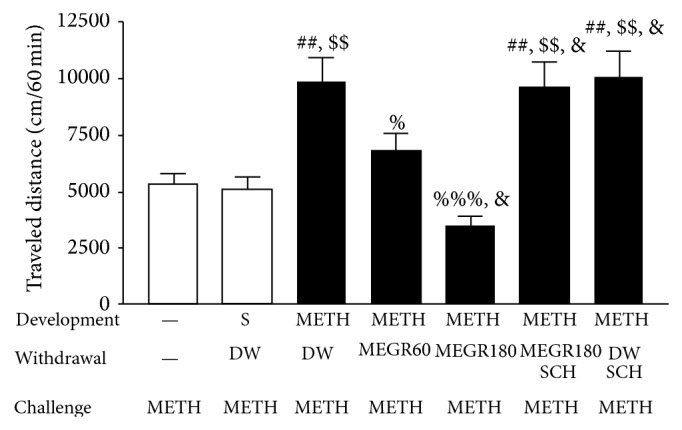
Effects of MEGR on METH-induced locomotor sensitization in rats. In this study, rats were given either saline or METH once a day for 6 days and then underwent 6 days of withdrawal; during the withdrawal period, either MEGR or DW was administered. The administration of METH over 6 consecutive days induced significant sensitization, which was attenuated by MEGR treatment. All data are expressed as means ± SEM (*n* = 7). MEGR: methanol extract of* G. radix*, DW: distilled water, S: saline, MEGR60: 60 mg/kg/day MEGR once a day for 6 consecutive days, MEGR180: 180 mg/kg/day MEGR once a day for 6 consecutive days, SCH: a single SCH50911 (3 mg/kg) treatment. ^##^ *P* < 0.01 versus a single METH group; ^$$^ *P* < 0.01 versus S/DW/METH group; ^%^ *P* < 0.05, ^%%%^ *P* < 0.001 versus METH/DW/METH group; ^&^ *P* < 0.05 versus METH/MEGR60/METH group (one-way ANOVA followed by Newman-Keuls post hoc test).

On the sixth day after the final dose of METH, a METH challenge produced a significantly larger increase in locomotor activity in METH-pretreated rats compared with saline-pretreated rats or rats that received only a challenge dose of METH (*F*(6,42) = 10.25, *P* < 0.0001; METH/DW/METH group (*n* = 7) versus saline/DW/METH group (*n* = 7), *q* = 5.69, *P* < 0.01; METH/DW/METH group versus METH group (*n* = 7), *q* = 5.45, *P* < 0.01). These data indicate that repeated exposure to METH induced the expression of locomotor sensitization. However, post hoc comparisons revealed that 60 and 180 mg/kg of MEGR significantly inhibited locomotor hypersensitivity (METH/DW/METH group versus METH/MEGR60/METH group (*n* = 7), *q* = 3.67, *P* < 0.05; METH/DW/METH group versus METH/MEGR180/METH group (*n* = 7), *q* = 7.63, *P* < 0.01) in a dose-dependent manner (METH/MEGR60/METH group versus METH/MEGR180/METH group, *q* = 3.97, *P* < 0.05). Post hoc comparisons also revealed that the inhibitory effects of 180 mg/kg of MEGR were blocked by pretreatment with SCH50911 (METH/MEGR180/METH group versus METH/SCH50911/MEGR180/METH group (*n* = 7), *q* = 7.35, *P* < 0.01). Pretreatment with SCH50911 alone, however, did not influence the expression of METH-induced locomotor sensitization, which indicates that the inhibitory effects of SCH50911 acted specifically against the effects of MEGR (Figure 3).

*G. radix* possesses sedative effects [14], and agents with sedative pharmacological actions inhibit spontaneous motor activity [20]. Thus, in an additional experiment, the effects of MEGR on locomotor activity were evaluated in rats receiving either a single injection or repeated injections of saline (in their home cages) on the same time schedule as the METH-induced locomotor sensitization paradigm. The administration of MEGR (180 mg/kg/day) over 6 days did not have an effect on the single saline-treated or the repeated saline-treated rats (data not shown). Additionally, there was no difference in locomotor activity between the single saline-treated rats and the repeated saline-treated rats (data not shown), indicating no development of locomotor sensitization in response to repeated saline injections in this procedure.

### 3.2. Effects of MEGR on the Development and Expression of METH-Induced CPP

The expression of locomotor sensitization following repeated exposure to METH was almost completely blocked by 180 mg/kg of MEGR. Thus, the same dose was used to examine the effects of MEGR on METH-induced CPP.

**Figure 4:**
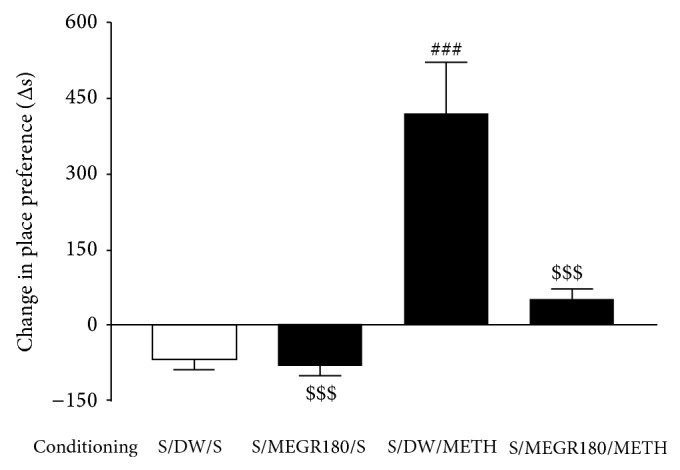
Effects of MEGR on the development of METH-induced CPP in rats. In this study, rats were administered either METH or saline on alternating days for 6 days, and MEGR or DW was given 60 min prior to every METH or saline injection in the nonpreferred chamber. METH treatment for 3 nonconsecutive days elicited CPP for the METH-paired chamber, which was blocked by pretreatment with MEGR. All data are expressed as means ± SEM (*n* = 7). MEGR: methanol extract of* G. radix*, DW: distilled water, S: saline, MEGR180: 180 mg/kg/day MEGR. _ _^###^ *P* < 0.001 versus S/DW/S group; ^$$$^ *P* < 0.001 versus S/DW/METH group (one-way ANOVA followed by Newman-Keuls post hoc test).

In the preconditioning phase, the rats showed a spontaneous preference for Chamber A (black walls and a smooth floor) over Chamber B (white walls and a textured floor), such that the mean time spent in Chamber A was 861.24 ± 43.76 s (*n* = 12), and the mean time spent in Chamber B was 214.87 ± 25.31 s (*n* = 12; *t* = 9.53, *P* < 0.0001, a paired *t*-test). Therefore, METH was administered while the rats were in Chamber B. One-way ANOVA and post hoc comparisons revealed that the rats receiving three pairings of METH (1 mg/kg, i.p.) with the naturally nonpreferred Chamber B spent a significantly greater amount of time in the METH-paired chamber compared with the saline-treated control group (*F*(3,24) = 18.92, *P* < 0.0001; saline/DW group (*n* = 7) versus METH/DW group (*n* = 7), *q* = 9.06, *P* < 0.001), indicating that METH-induced CPP was established. However, 180 mg/kg/day of MEGR given 60 min prior to every METH administration during the conditioning phase almost completely inhibited the development of METH-induced CPP (METH/DW group versus METH/MEGR180 group (*n* = 7), *q* = 6.83, *P* < 0.001), which demonstrates the deterrent effects of MEGR on the development of METH-induced CPP. Post hoc comparisons also revealed that MEGR treatment did not produce a place preference or aversion by itself (Figure 4).

**Figure 5:**
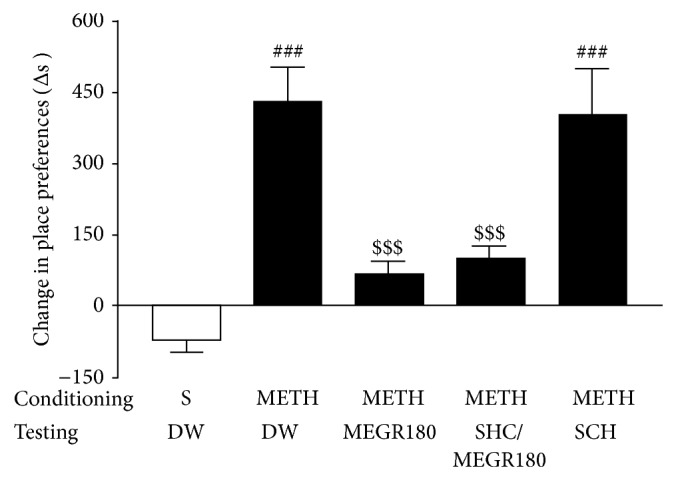
Effects of MEGR on the expression of METH-induced CPP in rats. In this study, rats were administered either METH or saline on alternating days for 6 days, and MEGR or DW was given 60 min prior to testing METH-induced CPP expression. METH treatment for 3 nonconsecutive days elicited CPP for the METH-paired chamber, which was blocked by pretreatment with MEGR. All data are expressed as means ± SEM (*n* = 7). MEGR: methanol extract of* G. radix*, DW: distilled water, S: saline, MEGR180: 180 mg/kg/day MEGR, SCH: a single SCH50911 (3 mg/kg) treatment, _ _^###^ *P* < 0.001 versus S/DW/S group; ^$$$^ *P* < 0.001 versus S/DW/METH group (one-way ANOVA followed by Newman-Keuls post hoc test).

Similarly, in the experiment evaluating the effect of MEGR on the expression of METH-induced CPP, a single administration of MEGR (180 mg/kg) 60 min prior to the testing phase significantly decreased the time the rats spent in the METH-paired chamber (*F*(4,30) = 15.31, *P* < 0.0001; saline/DW group (*n* = 7) versus METH/DW group (*n* = 7), *q* = 8.92, *P* < 0.001; METH/DW group versus METH/MEGR180 group (*n* = 7), *q* = 6.42, *P* < 0.001). These data demonstrate the inhibitory effects of MEGR on the expression of METH-induced CPP. However, prior treatment with SCH50911 (3 mg/kg) did not block these inhibitory effects of MEGR (180 mg/kg) (METH/DW group versus METH/SCH50911/MEGR180 group (*n* = 7), *q* = 5.86, *P* < 0.001), and SCH50911 treatment alone did not influence the expression of METH-induced CPP (Figure 5).

## 4. Discussion

Previous studies from our laboratory have demonstrated that MEGR and isoliquiritigenin suppress acute cocaine-induced accumbal DA release via GABAb receptors [16] and prevent acute METH-induced hyperlocomotion by inhibiting accumbal DA synthesis [17]. In the present study, we extended these findings, demonstrating that MEGR (60 or 180 mg/kg/day) dose-dependently inhibited repeated METH-induced locomotor sensitization, with nearly complete abolishment of sensitization induced by the 180 mg/kg dose. Moreover, this dose attenuated both the development and expression of METH-induced CCP effectively. In addition, the inhibitory effects of MEGR on the expression of METH-induced locomotor sensitization, but not the expression of CPP, were blocked by pretreatment with the selective GABAb receptor antagonist SCH50911.

Locomotor sensitization initially develops via the primary biological actions of acute METH. However, following repeated exposure to METH, this behavior is characteristic of various neural adaptations and more potent biological effects and plays a key role in the development of compulsive drug-seeking behaviors [21]. The expression of locomotor sensitization is markedly strengthened when tested in an environment (a cue or cues) that was previously associated with drug injections, because both the direct pharmacological action of METH and the learned associations with environmental cues contribute to the sensitization. As a result, interpretation of the effects of a particular medication on METH-induced locomotor sensitization becomes complicated [22]. To avoid this complication in the present study, METH and saline were administered unpaired with the locomotor testing box (the cue); thus, the pharmacological actions of repeated METH administration were uniquely responsible for the sensitization. This procedure appeared to be validated by the finding that repeated saline injections did not induce locomotor sensitization (Figure 3).

In the present study, the expression of repeated METH-induced locomotor sensitization was dose-dependently inhibited by MEGR (60 and 180 mg/kg/day), suggesting that MEGR exerts its preventative effects via blockade of the sensitizing pharmacological actions of METH. These data are compatible with previous studies showing that MEGR significantly inhibits acute cocaine-induced accumbal DA release via GABAb receptors [16] and acute METH-induced hyperlocomotion and accumbal DA synthesis [17], since the initial pharmacological effects of METH related to its rewarding properties are based on both the direct and indirect increases in accumbal DA. Additionally, the inhibitory effects of MEGR on the expression of METH-induced locomotor sensitization in the present study were blocked by the selective GABAb receptor antagonist SCH50911. Taken together, these results indicate that MEGR inhibits repeated METH-induced locomotor sensitization via GABAb receptors.

The CPP paradigm is another animal model widely used for measuring the rewarding effects of addictive drugs [23]. CPP is a form of classic Pavlovian conditioning in which a particular environment (an exteroceptive cue) is associated with METH exposure (an interoceptive cue). Therefore, the development and expression of CPP require the rewarding effects of METH in addition to the learned memory that associates METH with exteroceptive cues [24]. In the present CPP experiment, pretreatment with MEGR (180 mg/kg/day) almost completely abolished the development and expression of METH-induced CPP. Because the locomotor sensitization results demonstrated that MEGR can counteract the pharmacological action of METH related to reward, the inhibitory effects of MEGR on CPP may stem directly from its masking of interoceptive cues.

Meanwhile, in the experiment evaluating the effect of MEGR on the expression of METH-induced CPP, pretreatment with SCH50911 did not antagonize the inhibitory effects of MEGR on the expression of CPP, whereas in the locomotor sensitization model SCH50911 blocked the inhibitory effects of MEGR on the expression of pharmacological sensitization. This discrepancy implies that the inhibitory effects of MEGR on METH-induced CPP are mediated by both antagonizing the rewarding effects of METH and interfering with the association of interoceptive and exteroceptive cues, and that the latter is independent of the GABAb receptors. Taken together, these results suggest that MEGR effectively inhibits both the development and expression of METH-induced CPP and that the process may be mediated by blockade of the pharmacological actions of METH and/or the impedance of cue-associated learning and memory.

Metabotropic GABAb receptors are widely distributed throughout the brain and play a crucial role in regulating the reinforcing effects of psychostimulants [25]. The activation of GABAb receptors has an inhibitory effect on DA tone in both the VTA and NAcc. Baclofen, a GABAb receptor agonist, decreases cocaine-induced accumbal DA release [26], attenuates repeated cocaine-induced locomotor sensitization [27], and reduces cocaine and METH self-administration [28, 29]. Similarly, the suppressive effects of* G. radix* on acute cocaine-induced accumbal DA release are mediated via GABAb receptors [16]. The present study demonstrated that the effects of MEGR on repeated METH-induced locomotor sensitization were mediated through GABAb receptors, which indicates that this system may be an important pharmacological target for treating METH addiction. Additionally, the present findings indicate that MEGR inhibited METH-induced CPP by interfering with METH cue-associated memory, which is independent of GABAb receptors and implicates the involvement of other neurotransmitters. Given that glutamatergic receptors are critical in drug-cue associated memory processes [30] and that both isoliquiritigenin and glycyrrhizic acid antagonize the electrophysiological function of N-methyl-D-aspartate (NMDA) receptors to inhibit Ca2+ influx [31, 32], the glutamatergic system in the brain may be another important target for* G. radix* when treating METH addiction.

In summary, MEGR (60 or 180 mg/kg/day) dose-dependently attenuated the expression of METH-induced locomotor sensitization, and 180 mg/kg/day of MEGR blocked the development and expression of METH-induced CPP. Additionally, the effects of MEGR on locomotor sensitization, but not CPP expression, were abolished by pretreatment with the selective GABAb antagonist SCH50911. These data suggest that* G. radix* can block the development of METH dependence, which may be mediated by GABAb receptors.

## References

[1] Panenka W. J., Procyshyn R. M., Lecomte T., MacEwan G. W., Flynn S. W., Honer W. G., Barr A. M. (2013). Methamphetamine use: a comprehensive review of molecular, preclinical and clinical findings. *Drug and Alcohol Dependence*.

[2] Vocci F. J., Appel N. M. (2007). Approaches to the development of medications for the treatment of methamphetamine dependence. *Addiction*.

[3] Kosten T. A., Miserendino M. J. D., Chi S., Nestler E. J. (1994). Fischer and Lewis rat strains show differential cocaine effects in conditioned place preference and behavioral sensitization but not in locomotor activity or conditioned taste aversion. *Journal of Pharmacology and Experimental Therapeutics*.

[4] Häggkvist J., Lindholm S., Franck J. (2009). The effect of naltrexone on amphetamine-induced conditioned place preference and locomotor behaviour in the rat. *Addiction Biology*.

[5] Kurokawa K., Mizuno K., Shibasaki M., Higashioka M., Oka M., Hirouchi M., Ohkuma S. (2013). Acamprosate suppresses ethanol-induced place preference in mice with ethanol physical dependence. *Journal of Pharmacological Sciences*.

[6] di Chiara G., Imperato A. (1988). Drugs abused by humans preferentially increase synaptic dopamine concentrations in the mesolimbic system of freely moving rats. *Proceedings of the National Academy of Sciences of the United States of America*.

[7] de Rover M., Lodder J. C., Kits K. S., Schoffelmeer A. N. M., Brussaard A. B. (2002). Cholinergic modulation of nucleus accumbens medium spiny neurons. *European Journal of Neuroscience*.

[8] Podda M. V., Riccardi E., D'Ascenzo M., Azzena G. B., Grassi C. (2010). Dopamine D1-like receptor activation depolarizes medium spiny neurons of the mouse nucleus accumbens by inhibiting inwardly rectifying K+ currents through a cAMP-dependent protein kinase A-independent mechanism. *Neuroscience*.

[9] Erhardt S., Mathé J. M., Chergui K., Engberg G., Svensson T. H. (2002). GABAB receptor-mediated modulation of the firing pattern of ventral tegmental area dopamine neurons in vivo. *Naunyn-Schmiedeberg's Archives of Pharmacology*.

[10] Tyacke R. J., Lingford-Hughes A., Reed L. J., Nutt D. J. (2010). GABAB receptors in addiction and its treatment. *Advances in Pharmacology*.

[11] Wang Z. Y., Nixon D. W. (2001). Licorice and cancer. *Nutrition and Cancer*.

[12] Kim Y. W., Ki S. H., Lee J. R., Lee S. J., Kim C. W., Kim S. C., Kim S. G. (2006). Liquiritigenin, an aglycone of liquiritin in Glycyrrhizae radix, prevents acute liver injuries in rats induced by acetaminophen with or without buthionine sulfoximine. *Chemico-Biological Interactions*.

[13] Lee J. R., Park S. J., Lee H.-S., Jee S. Y., Seo J., Kwon Y. K., Kwon T. K., Kim S. C. (2009). Hepatoprotective activity of licorice water extract against Cadmium-induced toxicity in rats. *Evidence-Based Complementary and Alternative Medicine*.

[14] Gruenwald J. (2004). *PDR for Herbal Medicines*.

[15] Shishkina G. T., Dygalo N. N., Yudina A. M., Kalinina T. S., Tolstikova T. G., Sorokina I. V., Kovalenko I. L., Anikina L. V. (2006). The effects of fluoxetine and its complexes with glycerrhizic acid on behavior in rats and brain monoamine levels. *Neuroscience and Behavioral Physiology*.

[16] Jang E. Y., Choe E. S., Hwang M., Kim S. C., Lee J. R., Kim S. G., Jeon J.-P., Buono R. J., Yang C. H. (2008). Isoliquiritigenin suppresses cocaine-induced extracellular dopamine release in rat brain through GABAB receptor. *European Journal of Pharmacology*.

[17] Zhao Z., Wang Y., Lin F. (2014). Methanol extract from radix of *Glycyrrhizae uralensis* attenuate methamphetamine-induced hyperlocomotor activity. *Herbal Formula Science*.

[18] He Y., Tian X., Hu X., Porreca F., Wang Z. J. (2012). Negative reinforcement reveals non-evoked ongoing pain in mice with tissue or nerve injury. *he Journal of Pain*.

[19] Maeda T., Kiguchi N., Fukazawa Y., Yamamoto A., Ozaki M., Kishioka S. (2007). Peroxisome proliferator-activated receptor gamma activation relieves expression of behavioral sensitization to methamphetamine in mice. *Neuropsychopharmacology*.

[20] File S. E. (1984). Behavioural pharmacology of benzodiazepines. *Progress in Neuro-Psychopharmacology & Biological Psychiatry*.

[21] Miserendino M. J., Nestler E. J. (1995). Behavioral sensitization to cocaine: modulation by the cyclic AMP system in the nucleus accumbens. *Brain Research*.

[22] Badiani A., Robinson T. E. (2004). Drug-induced neurobehavioral plasticity: the role of environmental context. *Behavioural Pharmacology*.

[23] Tzschentke T. M. (1998). Measuring reward with the conditioned place preference paradigm: a comprehensive review of drug effects, recent progress and new issues. *Progress in Neurobiology*.

[24] Mucha R. F., Iversen S. D. (1984). Reinforcing properties of morphine and naloxone revealed by conditioned place preferences: a procedural examination. *Psychopharmacology*.

[25] Vlachou S., Markou A. (2010). GABAB receptors in reward processes. *Advances in Pharmacology*.

[26] Fadda P., Scherma M., Fresu A., Collu M., Fratta W. (2003). Baclofen antagonizes nicotine-, cocaine-, and morphine-induced dopamine release in the nucleus accumbens of rat. *Synapse*.

[27] Frankowska M., Nowak E., Filip M. (2009). Effects of GABAB receptor agonists on cocaine hyperlocomotor and sensitizing effects in rats. *Pharmacological Reports*.

[28] Brebner K., Phelan R., Roberts D. C. S. (2000). Intra-VTA baclofen attenuates cocaine self-administration on a progressive ratio schedule of reinforcement. *Pharmacology Biochemistry and Behavior*.

[29] Ranaldi R., Poeggel K. (2002). Baclofen decreases methamphetamine self-administration in rats. *NeuroReport*.

[30] Peters J., de Vries T. J. (2012). Glutamate mechanisms underlying opiate memories. *Cold Spring Harbor Perspectives in Medicine*.

[31] Kawakami Z., Ikarashi Y., Kase Y. (2011). Isoliquiritigenin is a novel NMDA receptor antagonist in kampo medicine yokukansan. *Cellular and Molecular Neurobiology*.

[32] Cherng J. M., Lin H. J., Hung M. S., Lin Y. R., Chan M. H., Lin J. C. (2006). Inhibition of nuclear factor *κ*B is associated with neuroprotective effects of glycyrrhizic acid on glutamate-induced excitotoxicity in primary neurons. *European Journal of Pharmacology*.

